# A HIF-1α inhibitor combined with palmitic acid and L-carnitine treatment can prevent the fat metabolic reprogramming under hypoxia and induce apoptosis in hepatocellular carcinoma cells

**DOI:** 10.1186/s40170-023-00328-w

**Published:** 2023-12-08

**Authors:** Shohei Matsufuji, Yoshihiko Kitajima, Kazuki Higure, Naoya Kimura, Sachiko Maeda, Kohei Yamada, Kotaro Ito, Tomokazu Tanaka, Keita Kai, Hirokazu Noshiro

**Affiliations:** 1 Department of Surgery, Saga University Faculty of Medicine, Saga, 849-8501 Japan; 2 Department of Surgery, National Hospital Organization, Higashisaga Hospital, Saga, Miyaki 849-0101 Japan; 3 Department of Pathology, Saga University Faculty of Medicine, Saga, 849-8501 Japan

**Keywords:** HCC, HIF-1α, Hypoxia, Fatty acid oxidation, YC-1, Palmitic acids, L-carnitine, ROS

## Abstract

**Background:**

A hypoxic environment often persists within solid tumors, including hepatocellular carcinoma (HCC). Hypoxia-inducible factor-1α (HIF-1α) can accelerate cancer malignancy by inducing hypoxia-dependent expression of various genes. Tumor hypoxia can also induce metabolic reprogramming of fatty acid (FA) metabolism, through which HIF-1α plays an essential role in diminishing fatty acid β-oxidation (FAO) in hypoxic cancer cells.

**Methods:**

We aimed to investigate potential new drug therapy options for targeting hypoxic cancer cells within HCC tumors, specifically through combining HIF-1α inhibition with palmitic acid (PA) + L-carnitine (LC) treatment to effectively induce apoptosis in hypoxic HCC cells. To test this hypothesis, in vitro and in vivo studies were performed.

**Results:**

We first demonstrated that hypoxia-dependent apoptosis was induced by an overload of PA in two HCC cell lines (HepG2 and Hep3B) via excessive production of reactive oxygen species (ROS). Moreover, this observed PA-induced apoptosis was enhanced by HIF-1α knockdown (KD) in these cells under hypoxia. In addition, the combination of PA with FAO activator LC increased FAO activity and led to stronger cell death than PA alone in hypoxic HIF-1α KD cells, specifically through further ROS generation. To clarify the mechanism of hypoxia-induced FA metabolism reprogramming, expression levels of the genes encoding FAO enzymes CPT1A, ACSL1, MCAD, and LCAD, FA transporter CD36, and FA esterification enzymes DGAT and APGAT were analyzed using HIF-1α KD and scramble control (SC) cells. The results suggested that HIF-1α could repress mRNA expression of the FAO-related enzymes and CD36, while it upregulated FA esterification gene expression. This suggested a central role for HIF-1α in hypoxia-induced reprogramming of FA metabolism in HCC cells. Using a nude mouse model, PA administration was found to induce apoptosis from ROS overproduction in HIF-1α KD tumors compared with SC tumors. Additional LC treatment synergistically enhanced the PA-induced apoptosis in HIF-1α KD tumors. Finally, in vivo therapy composed of HIF-1α inhibitor YC-1 with PA + LC could induce ROS-mediated apoptosis in HepG2 tumors without significant toxicity.

**Conclusions:**

A combination therapy of YC-1 with PA + LC may be a unique anti-tumor therapy for targeting hypoxic HCC cells, specifically by ROS overproduction leading to forced FAO activation.

**Supplementary Information:**

The online version contains supplementary material available at 10.1186/s40170-023-00328-w.

## Background

Liver cancer is the sixth most common cancer worldwide and the fourth leading cause of cancer-related death [1]. Hepatocellular carcinoma (HCC) is the major histological type of primary liver cancer, accounting for 75% of cases [2]. Recently, alcoholic liver disease and nonalcoholic fatty liver disease (NAFLD) and nonalcoholic steatohepatitis (NASH), in addition to hepatitis B virus (HBV) or HCV infection, have been found to be risk factors for developing HCC [3, 4]. Hepatic resection or ablation is recommended as the primary treatment method for HCC when liver function is preserved [4]. However, HCC has a high rate of recurrence, even after locally curative treatment. After resection, the 5-year survival rate exceeds 60%, while 70% of these patients have a recurrent tumor over the next 5 years [5, 6]. Postoperative adjuvant therapy after radical hepatectomy reportedly showed little improvement in patient recurrence-free survival (RFS) rates [7, 8]. In recent years, molecular targeted drugs, including sorafenib, regorafenib, and bevacizumab, as well as immunotherapy drugs such as atezolizumab, have been widely approved for HCC treatment [9–13]. Unfortunately, these approaches are still insufficient for improving HCC patient survival rates. Therefore, novel drug therapies need to be developed to increase these rates.

Hypoxia is closely associated with the progression of solid tumors, including HCC [14]. The transcription factor hypoxia-inducible factor 1α (HIF-1α) is a central mediator of tumor hypoxia [15]. Under normoxia, prolyl hydroxylases (PHDs) use oxygen as a substrate to hydroxylase key proline residues within HIF-1α, which is then degraded through the proteasomal pathway [16]. Under hypoxia, however, PHD activity is inhibited, allowing HIF-1α protein to be stabilized and form a complex with the constitutively expressed HIF-1β. The HIF-1α/HIF-1β heterodimer (HIF-1) binds to hypoxia response elements (HREs) in the promoter regions of various target genes [17–19]. To date, HIF-1α expression has been identified as a poor prognostic factor for patients with various malignancies, including HCC [20–27]. Recently, numerous studies have reported the essential roles of HIF-1α in metabolic reprogramming in hypoxic cancer cells [18]. In glucose metabolism, HIF-1 induces the metabolic switch from mitochondrial respiration to anaerobic glycolysis under hypoxia [18]. Previous studies reported that HIF-1α upregulates expression of critical genes, which promoting anaerobic glycolysis such as glucose transporter GLUT1, glycolytic enzymes, pyruvate dehydrogenase kinase 1 (PDK1) and lactate dehydrogenase A (LDHA) [15, 18, 28, 29]. In addition, mitochondrial respiration under hypoxia is known to produce reactive oxygen species (ROS) [30]. Intracellular ROS are mainly generated through mitochondrial oxidative phosphorylation (OXPHOS), a process performed by the electron transport chain (ETC) [31]. A recent study revealed evidence that mitochondrial complex I deactivation of OXPHOS is a critical mechanism of ROS production under acute hypoxia [30]. When ROS overwhelm the cellular antioxidant defense system, oxidative stress occurs. Excessive oxidative stress can result in ROS-mediated damage of nucleic acids, proteins, and lipids, leading to cell death [32]. Hence, anaerobic glycolysis, which is regulated by HIF-1, is an adaptive strategy for cancer cells to avoid mitochondrial respiration and survive under hypoxic conditions [33].

Recently, accumulating studies have reported that hypoxia can also alter lipid metabolism in cancer cells [34]. Previous work has described that HIF-1 can regulate metabolic reprogramming in fatty acid b-oxidation (FAO) and lipid accumulation in cancer cells under hypoxia [35–37]. Additionally, one study reported that treatment of HepG2 cells with 1 mM palmitic acid (PA), a long-chain saturated fatty acid with 16 carbons, induced apoptosis under hypoxia, but not under normoxia [38]. The authors also demonstrated that this PA-induced lipo-apoptosis was mediated by excessive ROS production [38]. However, this study presented PA-induced lipo-apoptosis under hypoxia as a pathogenic mechanism of disease progression from NAFLD to NASH occurring in patients with obstructive sleep apnea [38].

From the previous reports, we hypothesized that PA-induced lipo-apoptosis may possibly be further enhanced by inhibiting HIF-1α expression in HCC cells under hypoxia, thereby abrogating the reprogramming of lipid metabolism and producing more ROS. We also anticipated that this would lead to a novel therapeutic concept that PA treatment combined with HIF-1 inhibition could induce apoptosis specifically in the hypoxic cancer cells of HCC tumors.

In the present study, we first uncovered the precise mechanism underlying PA-induced lipo-apoptosis in hypoxia using the HCC cell lines HepG2 and Hep3B. Next, we evaluated if HIF-1 inhibition could increase cell apoptosis rates with PA treatment in the hypoxic HCC cells. Furthermore, we assessed whether a FAO activator L-carnitine (LC) could enhance the PA-induced apoptosis. Finally, we investigated the in vivo effects of combination treatment by YC-1 with PA + LC on tumor growth in nude mice. Overall, the goal of this study was to establish an in vivo model of a novel drug therapy that can target hypoxic cancer cells within HCC tumors.

## Methods

### Cell lines

The human HCC cell line HepG2 was obtained from the Japanese Cancer Research Resources Bank (Osaka, Japan), while Hep3B cells were obtained from the American Type Culture Collection (ATCC, Manassas, VA, USA). Cells were cultured in DMEM supplemented with 10% fetal bovine serum and 100 μg/mL kanamycin and incubated at 37 °C in a humidified atmosphere. Cells were cultured under either normoxic conditions (20% O_2_ and 5% CO_2_) or hypoxic conditions (1% O_2_, 5% CO_2_, and 94% N_2_). Cells were transfected with a plasmid containing a short hairpin RNA (shRNA) targeting HIF-1α or a scrambled (control) shRNA. Additionally, a small interfering RNA (siRNA) targeting CPT1A or scrambled control siRNA was transiently transfected into cells. Dimethyloxalylglycine (DMOG; 1 mM) was used as the PHD inhibitor to mimic hypoxic conditions and N-acetyl-L-cysteine (NAC; 5 mM) was used as the ROS scavenger.

### shRNA and siRNA sequences

The pBAsi-hU6 Pur DNA plasmid (Takara, Shiga, Japan) was used to construct a HIF-1α shRNA plasmid. The shRNAs were designed as follows: HIF-1α (5′-CCACATTCA CGTATATGAT-3′) and scrambled (5′-CAACAAGATGAA GAGCACCAA-3′). Cells were transfected with the plasmid encoding HIF-1α or scrambled control for 72 hours using Lipofectamine® 3000 (Thermo Fisher Scientific, Waltham, MA, USA) according to the manufacturer’s instructions. Selection of transfected cells was performed using puromycin (1.5 mg/mL in culture medium) and harvested 7–10 days after transfection, as previously described [23].

The CPT1A siRNA (ON-TARGETplus Human CPT1A siRNA) and control siRNA (ON-TARGETplus Non-targeting Control siRNA) was purchased from Dharmacon (Cambridge, UK). Transfection of siRNAs was performed using Lipofectamine RNAiMAX® (Thermo Fisher Scientific) according to the manufacturer’s instructions. Transfected cells ware used for experiments within 5 days.

### Real-time quantitative polymerase chain reaction (qRT-PCR)

Total RNA was isolated from cells after culturing for 24 hours under normoxic or hypoxic conditions. Total RNA was reverse transcribed to cDNA, then qRT-PCR was performed, as described previously [25]. The mRNA levels were normalized to β-actin expression. All experiments were performed in triplicate. The primers used for qRT-PCR were as follows: CPT1A: 5′- ATC AAT CGG ACT CTG GAA ACG G − 3′ (forward), 3′- TCA GGG AGT AGC GCA TGG T − 5′ (reverse); ACSL1: 5′- CGA CGA GCC CTT GGT GTA TTT − 3′ (forward), 3′- GGT TTC CGA GAG CCT AAA CAA − 5′ (reverse); MCAD: 5′- TGG GAG GTT GAT TCT GGT GGT CG − 3′ (forward), 3′- TGT CAA TGT GTT CAC GGG CT − 5′ (reverse); LCAD: 5′- TTG GCA AAA CAG TTG CTC AC − 3′ (forward), 3′- ACA TGT ATC CCC AAC CTC CA − 5′ (reverse); CD36: 5′- TGG GTT AAA ACA GGC ACA GA − 3′ (forward), 3′- CAG CGT CCT GGG TTA CAT TT − 5′ (reverse); DGAT1: 5′- TCG CCT GCA GGA TTC TTT AT − 3′ (forward), 3′- AAG ACA TTG GCC GCA ATA AC − 5′ (reverse); APGAT1: 5′- GAG GGA ACG AGA AAC CAC AA − 3′ (forward), 3′- GAC ATT GTC CCG AGG TGA AG − 5′ (reverse); ACTB: 5′- ACG CCT CTG GCC GTA CCA CT − 3′ (forward), 3′- TAA TGT CAC GCA CGA TTT CCC − 5′ (reverse).

### Western blot analysis

Whole cell lysates were harvested from cells cultured under normoxic or hypoxic conditions for 48 hours and from xenograft tumors in mice in lysis buffer and protease inhibitor cocktail mix. Western blot analysis was performed as described previously [25]. Primary antibodies: HIF-1α (1:1000; cat. no. 610959; BD Biosciences, California, USA), cleaved caspase 3 (1:500; cat. no. 5625; Cell Signaling Technology, Massachusetts, USA), cleaved PARP (1:1000; cat. no. 9661; Cell Signaling Technology), CPT1A (1:1000; cat. no. 12252; Cell Signaling Technology), MCAD (1:1000; cat. no. 55210–1-AP; Proteintech, Illinois, USA), LCAD (1:2000; cat. no. 17442–1-AP; Proteintech), ACSL1 (1:1000; cat. no. 13989–1-AP; Proteintech), AGPAT1 (1:1000; cat. no. 10601–1-AP; Proteintech), DGAT1 (1:1000; cat. no. 11561–1-AP; Proteintech), and CD36 (1:1000; cat. no. 18836–1-AP; Proteintech). Actin (1:10,000, cat. no. AC15; Sigma-Aldrich; Merck KGaA, Missouri, USA) was used as a loading control.

### Cell proliferation assay

Cell proliferation was assessed by trypan blue dye exclusion assays. Cells were treated with PA and LC and incubated for 48 hours under normoxia or hypoxia. These cells were trypsinized and counted using a TC20 cell counter (Bio-Rad, Calofornia, USA). All experiments were performed in triplicate and independently repeated at least three times. In addition, cell proliferation was also measured by 3-(4,5-dimethylthiazol-2-yl)-5-(3-carboxymethoxyphenyl)-2-(4-sulfophenyl)-2H-tetrazolium, inner salt (MTS) assays using the CellTiter 96® Aqueous One Solution Cell Proliferation Assay Kit (Promega, Madison, WI, USA) according to the manufacturer’s protocols.

### ROS detection analysis

ROS values were evaluated using a Total ROS Detection Kit (Enzo Life Science, New York, USA). Briefly, cells treated with PA and L-carnitine were incubated for 48 hours under normoxia or hypoxia. Cells were harvested and stained with ROS detection solution according to the manufacturer’s protocols. ROS fluorescence was detected using a FACS Verse flow cytometer (BD Biosciences), and data analysis was performed using FlowJo 10.7.1 (BD Biosciences). Dead cells were removed using 7-Aminoactinomycin D (BD Biosciences).

### FAO analysis

FAO was evaluated using FAO-Blue reagent (Funacoshi, Tokyo, Japana). To mimic hypoxic conditions, cells were treated with DMOG under normoxia for 12 hours. Thereafter, the culture medium was removed and FAO-Blue (final concentration, 10 μM) was added for 60 minutes. After this incubation, blur fluorescence (excitation 405 nm/emission 430 nm) was observed and quantified. Cell fluorescence was evaluated using LSM880 Airyscan (Carl *Zeiss* Co., Oberkochen, Germany*)*. Data were analyzed by ZEN (Carl *Zeiss* Co.).

### Flow cytometric analysis of apoptosis

Apoptosis rates were evaluated using an Annexin V-FITC Apoptosis Detection Kit (BioVision, Massachusetts, USA). Cells were cultured for 48 hours under normoxia or hypoxia with various drug treatments. Harvested cells were washed with binding buffer and then incubated with Annexin V-FITC and propidium iodide for 5 minutes. Fluorescence was analyzed using a FACS Verse flow cytometer (BD Biosciences). Apoptosis rates were estimated by the sum of Annexin V (−)/PI (+) and Annexin V (+)/PI (+).

### Oil red staining

Oil red staining of intracellular lipid droplets was performed using the Steatosis Fluorometric assay kit (Cayman Chemical, Michigan, USA). Cells were cultured under normoxia or hypoxia with or without 50 μM PA for 48 hours. Cells were then stained with oil red according to the manufacturer’s protocols. Stained lipid droplets were assessed by BZ-X800 microscopy (KEYENCE, Co., Osaka, Japan) and quantified. Quantification experiments were performed in triplicate. The optical density (OD) of each sample was measured at 570 nm in a microplate reader according to the manufacturer’s protocols.

### In vivo nude mice study

The animal experiments were approved by the Animal Care Committee of Saga University (Saga, Japan; Approval No. A2021–036-0). Four-week-old female BALB/c nude mice were obtained from Nihon Crea Co. (Tokyo, Japan). They were given sterile food and water under specific pathogen-free conditions. Body weight and tumor volume were measured twice per week. Tumor volume (T) was calculated as follows. T = π/4 x *a* x *b,* where *a* is the shorter axis (mm) and *b* is the longer axis (mm). Subcutaneous xenograft tumors were generated by injection of 3 × 10^6^ HepG2-SC, HepG2-HIF1KD, or HepG2 wild-type cells into both flanks of the nude mice. These mice were euthanized 14 to 18 days after drug administration, or once the tumor diameter reached more than 20 mm or the sum of right and left tumor diameters reached more than 30 mm. If body weight loss was more than 20%, the mouse was euthanized immediately regardless of tumor size.

### Palmitic acid, L-carnitine, and YC-1 injections

In experiments with the HIF-1α KD and SC cell lines, the mice were separated into four groups (control; *n* = 5, PA; n = 5, LC; n = 5, PA and LC; n = 5). Ten days after subcutaneous inoculation of the KD and SC cells, these mice were intraperitoneally (i.p.) injected daily with PA (50 mg/kg/day), LC (200 mg/kg/day), both PA and LC, or vehicle (300 μL 7.5% BSA and NaOH solution and 100 μL saline). In another experiment using YC-1, mice were separated into four groups (control; *n* = 5, YC-1; *n* = 4, PA + LC; n = 5, YC-1 with PA + LC; n = 5). YC-1 was also i.p. administered at 5 mg/kg daily.

### PA solution preparation method

Palmitate/BSA solution was made as described previously [39]. Firstly, 100 mM palmitic stock solution was prepared with 0.1 M NaOH warmed at 70 °C in a shaking water bath for 10 minutes. For in vitro experiments, the palmitic stock solution was diluted at a 1:20 volume ratio with 10% BSA solution at 55 °C in a water bath, then immediately vortexed. The complex solution was diluted in DMEM at a concentration ranging from 50 μM to 200 μM. For in vivo experiments, the palmitic stock solution was diluted at a 1:3 volume ratio. The mice were injected with 50 mg/kg PA.

### Plasma FFA measurement

The mice were euthanized and blood was collected 4 hours after palmitate solution administration. The blood was collected by cardiac puncture and centrifuged at 3000×g for 10 minutes at 4 °C, then plasma was collected and stored at − 80 °C until analysis. Free fatty acids (FFAs) in plasma samples were measured using the Free Fatty Acid Assay Kit (BioAssay Systems, California, USA). The OD values of the 10 μL of Plasma and Reaction Mix samples were measured at 570 nm in a microplate reader. All samples were examined in triplicate.

### Statistical analysis

All data are expressed as the mean ± standard error of the mean (SEM). For comparative analysis between two groups, data were analyzed using Student’s t-tests. For multiple group comparisons, one-way ANOVA followed by Tukey’s post hoc test was used. *P*-values < 0.05 were considered statistically significant. All data were analyzed using JMP Pro 16 software (SAS Institute, Inc., North Carolina, USA).

## Results

### PA could induce lipo-apoptosis in HCC cell lines under hypoxia

**Fig. 1:**
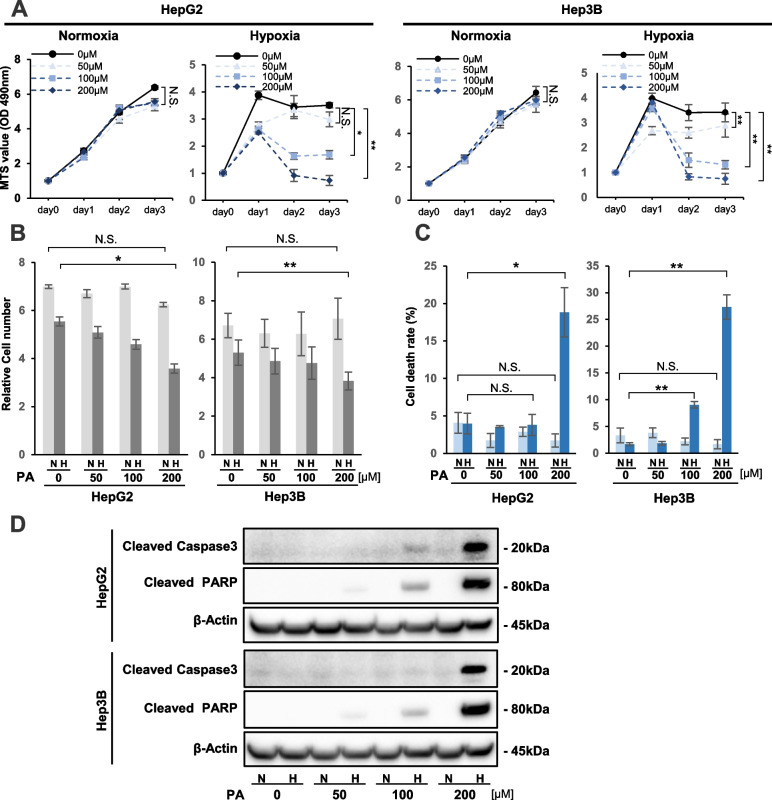
Effect of palmitic acid (PA) treatment on cell proliferation in HCC cells under hypoxia. **A** Viability of PA-treated HepG2 and Hep3B cells under normoxia and hypoxia by MTS assays. Cells were treated with PA at 0, 50, 100, and 200 μM concentrations. MTS values were estimated up to 72 hours of culture under normoxia and hypoxia. **B**,** C** Relative cell number of PA-treated HepG2 and Hep3B cells and trypan blue exclusion assay. For each cell line, 5.0 × 10^4^ cells were treated with PA at 0, 50, 100, and 200 μM concentrations under normoxia (N) and hypoxia (H) for 48 hours. **B** Living cell number and **C** cell death rate are plotted. **D** Western blot analysis of apoptosis-related protein expression, including cleaved caspase 3 and cleaved PARP, in HepG2 and Hep3B cells that were treated with PA at the indicated concentration under hypoxia for 48 hours. Values are presented as the mean ± standard error of the mean (SEM) of three independent experiments (**A**,** B**,** C**). N.S.: not significant, *P < 0.05, **P < 0.01 versus control

We first evaluated the effect of PA on cell viability in HepG2 and Hep3B cells by MTS assays under normoxic (20% O_2_) and hypoxic (1% O_2_) conditions (Fig. 1A). Under normoxic conditions, the viabilities of both HCC cell lines were not different between the control cells and cells treated with PA at 50, 100, and 200 μM concentrations (Fig. 1A). In contrast, under hypoxia, PA treatment remarkably decreased the viability of the two cell lines in a dose-dependent manner (Fig. 1A). Cell count analysis also showed that PA treatment decreased cell proliferation rates with dose dependency in both cell lines specifically under hypoxia (Fig. 1B). Next, the effects of PA treatment on cell death rates were estimated under normoxic and hypoxic conditions (Fig. 1C). The data indicated significantly elevated death rates in both cell lines with 200 μM PA treatment compared with no treatment under hypoxic conditions, whereas cell death was not significantly affected by PA treatment in HCC cells under normoxic conditions (Fig. 1C). Western blot (WB) analysis of apoptotic markers showed that the protein expression levels of cleaved caspase 3 and cleaved PARP were increased in both cell lines by PA treatment with dose dependency under hypoxia, but not normoxia (Fig. 1D). Flow cytometric analysis was also used to evaluate cell apoptosis (Supplementary Fig. S1). Levels of apoptosis were increased by PA treatment with dose dependency in the two cell lines under hypoxia (Supplementary Fig. S1).

### PA-induced lipo-apoptosis was triggered by excess ROS production through the FAO pathway under hypoxia

**Fig. 2:**
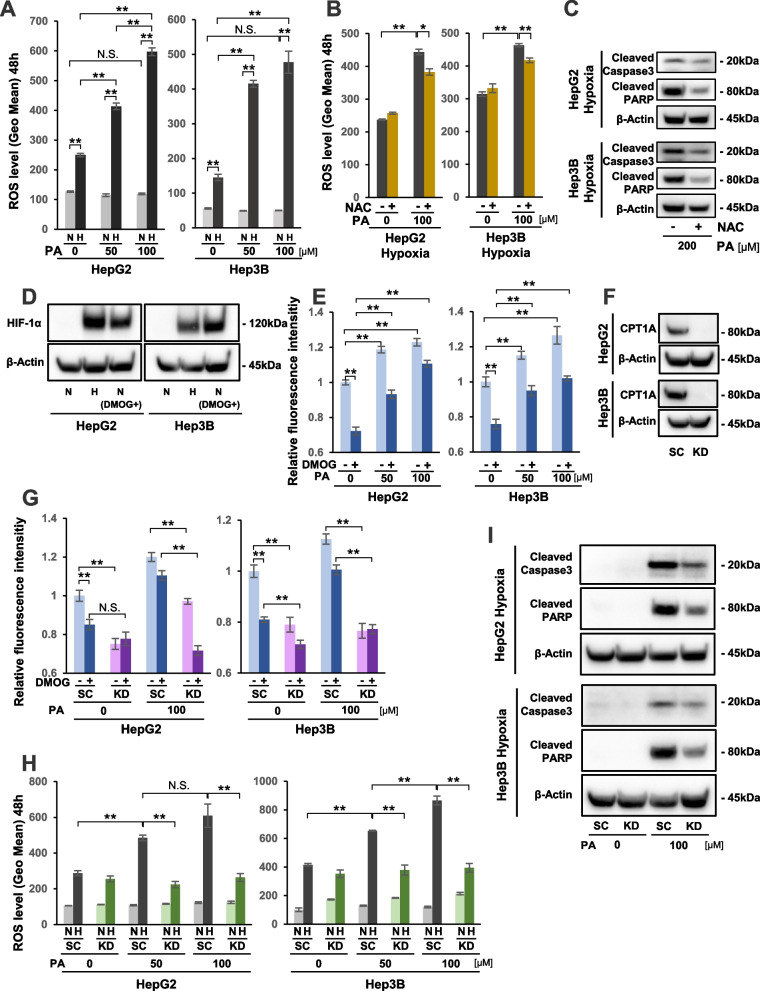
ROS production via the FAO pathway could induce apoptosis in HCC cells treated with PA. **A** ROS levels in HCC cells with or without 50/100 μM PA treatment for 48 hours. N: normoxia, H: hypoxia. **B** ROS levels in 100 μM PA-treated or untreated HCC cells under hypoxia with or without 1 mM N-acetyl-L-cysteine (NAC) treatment for 48 hours. **C** Western blot analysis of cleaved caspase 3 and cleaved PARP protein expression in 200 μM PA-treated HCC cells under hypoxia with or without 5 mM NAC treatment. β-actin expression was analyzed as an internal control. **D** Western blot analysis of HIF-1α protein expression in HCC cells under normoxia (N) and hypoxia (H) for 12 hours. In parallel, HIF-1α expression was analyzed in HCC cells that were treated with 1 mM dimethyloxalylglycine (DMOG) under normoxia, indicated as N (DMOG+). β-actin expression was analyzed as an internal control. **E** Using the FAO Blue system, in vitro FAO activity was analyzed in HCC cells that were treated with PA under normoxic (DMOG-) and hypoxia mimicking (DMOG+) conditions. **F** Western blot analysis of CPT1A protein expression in CPT1A knockdown (KD) and scramble control (SC) HCC cells. **G** FAO activity in KD and SC cells with or without 100 μM PA treatment under normoxic (DMOG-) and hypoxia mimicking (DMOG+) conditions. **H** ROS levels in KD and SC cells under normoxia (N) and hypoxia (H) with or without PA treatment for 48 hours. **I** Western blot analysis of cleaved caspase 3 and cleaved PARP protein expression in KD and SC cells with or without 100 μM PA treatment under hypoxia. β-actin expression was analyzed as an internal control. Values are presented as the mean ± standard error of the mean (SEM) of three independent experiments (**A**,** B**,** E**,** G**,** H**). N.S.: not significant, *P < 0.05, **P < 0.01 versus control

To elucidate the mechanism of PA-induced apoptosis in hypoxic HepG2 and Hep3B cells, we investigated intracellular ROS production in the PA-treated HCC cells (Fig. 2). As shown in Fig. 2A, ROS levels were significantly elevated in both cell lines under hypoxia compared with under normoxia, even without PA treatment. The ROS levels were further increased by PA treatment in a dose-dependent manner under hypoxic conditions, whereas ROS production was not influenced by PA under normoxia in either cell line (Fig. 2A). Next, we aimed to clarify if hypoxia-induced ROS production could directly cause apoptosis in PA-treated HCC cells. We first confirmed that the elevated ROS production following 100 μM PA treatment in hypoxic HCC cells was significantly reduced by using the antioxidant agent NAC at 1 mM (Fig. 2B). Next, we showed that cleaved caspase 3 and cleaved PARP protein expression levels, which were elevated with 200 μM PA treatment under hypoxia, were decreased with the addition of 5 mM NAC (Fig. 2C). These results suggested that the PA-induced lipo-apoptosis in hypoxic HCC cells was caused by excess ROS production. Furthermore, to clarify if the lethal ROS production was mediated via the FAO pathway, we performed the in vitro FAO-Blue assay on HCC cells under hypoxia (Fig. 2D, E). To induce HIF-1α expression even under normoxia, cells were treated with PHD inhibitor DMOG to mimic a hypoxic environment (Fig. 2D). As shown in Fig. 2E, FAO activity without PA treatment was significantly higher in both cell lines under normoxia (DMOG-) than in hypoxia mimicking (DMOG+) conditions, suggesting that FAO activity is suppressed under hypoxia. The FAO activity was significantly increased by PA treatment with dose dependency under normoxia. Interestingly, even in hypoxia-mimicking conditions, PA treatment resulted in higher FAO activity in both HCC cell lines (Fig. 2E). These findings led us to hypothesize that the lethal ROS were generated through elevated FAO pathway activity in PA-treated HCC cells under hypoxia. Moreover, we performed siRNA-mediated knockdown (KD) of the CPT1A gene, which encodes the rate limiting enzyme in the FAO pathway. This was to determine if inhibiting the FAO pathway would lead to reduced ROS production and subsequent suppression of apoptosis by PA treatment in hypoxic HCC cells. As shown in Fig. 2F, we confirmed that siRNA transfection resulted in strong inhibition of CPT1A expression in both cell lines. FAO analysis indicated that, regardless of PA treatment, CPT1A KD cells showed significantly suppressed FAO activity under normoxic (DMOG-) conditions compared with control SC cells (Fig. 2G). Furthermore, the FAO activity in CPT1A KD cells with 100 μM PA treatment was remarkably decreased under hypoxia mimicking (DMOG+) conditions compared with SC cells (Fig. 2G). The dose-dependent elevation of ROS production, which was observed in hypoxic SC cells treated with 50 μM to 100 μM PA, was strongly suppressed in hypoxic CPT1A KD cells (Fig. 2H). Finally, protein expression levels of cleaved caspase 3 and cleaved PARP, which were increased following 100 μM PA treatment in hypoxic SC cells, were apparently suppressed in KD cells under hypoxia (Fig. 2I). These results clearly showed that lipo-apoptosis, which was induced in hypoxic HCC cells following PA treatment, occurred through the activated FAO pathway.

### HIF-1α knockdown further activated the FAO pathway and led to stronger apoptosis in PA-treated HCC cells under hypoxia

**Fig. 3:**
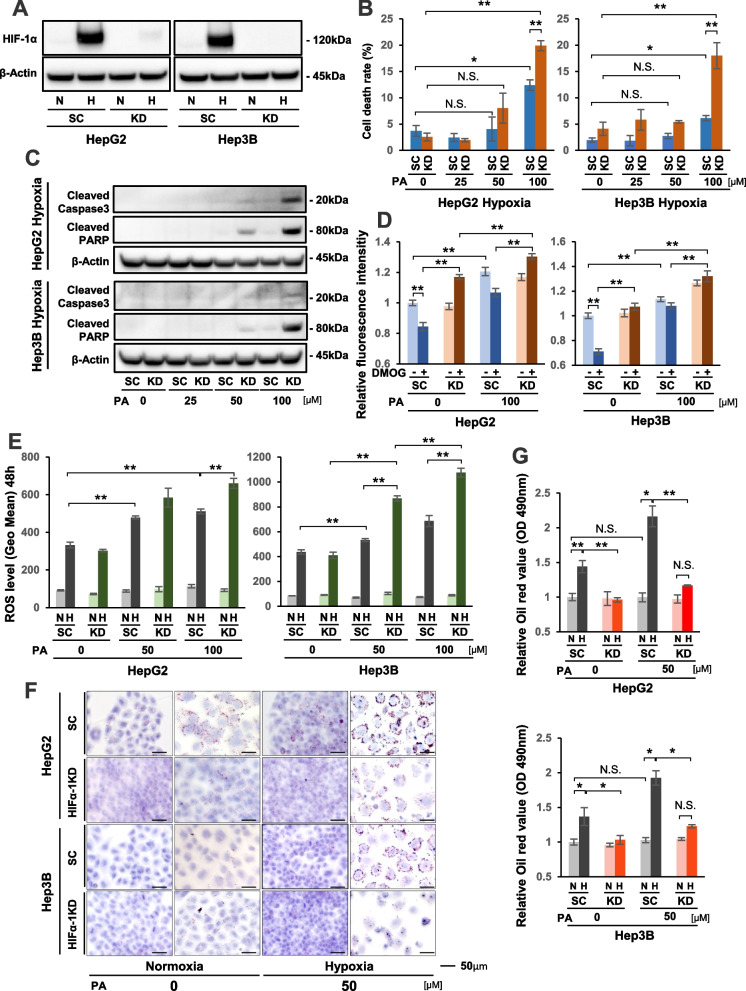
Effect of HIF-1α knockdown (KD) on lipo-apoptosis in HepG2 and Hep3B cells under hypoxia. **A** Western blot analysis of HIF-1α protein expression in HIF-1α KD and scramble control (SC) HepG2 and Hep3B cells under normoxia (N) and hypoxia (H) for 24 hours. **B** Cell death rates in KD and SC HepG2 and Hep3B cells with palmitic acid (PA) treatment at 0, 25, 50, and 100 μM concentrations under normoxia and hypoxia for 48 hours. **C** Western blot analysis of cleaved caspase 3 and cleaved PARP protein expression in KD and SC cells that were treated with PA at 0–100 μM concentrations under hypoxia for 48 hours. **D** Fatty acid β-oxidation (FAO) activity in KD and SC cells with or without 100 μM PA treatment under normoxia (DMOG-) and hypoxia mimicking (DMOG+) conditions for 12 hours. **E** Reactive oxygen species (ROS) levels in KD and SC cells under normoxia (N) and hypoxia (H) with PA (0–100 μM) for 48 hours. **F**,** G** Evaluation of fat deposition in KD and SC cells. Oil red staining was performed under normoxia (N) and hypoxia (H) with 50 μM PA treatment for 24 hours. Scale bars, 50 μm. Data are expressed as the mean ± standard error of the mean (SEM) (**B**,** D**,** E**,** G**). N.S.: not significant, *P < 0.05, **P < 0.01 versus control

To evaluate the biological effects of HIF-1 on lipo-apoptosis in HCC cells under hypoxia, we established stable HIF-1a KD HepG2 and Hep 3B cell lines (Fig. 3A). As shown in Fig. 3B, cell death rates were significantly increased by 100 μM PA treatment in SC and KD cells of both HCC cell lines under hypoxia compared with cells receiving no treatment. Furthermore, cell death rates were significantly higher in KD cells than SC cells with 100 μM PA under hypoxia (Fig. 3B). Western blot analysis showed stronger expression patterns of apoptosis-related proteins in KD cells than in SC cells following 100 μM PA treatment under hypoxia (Fig. 3C). Flow cytometric analysis also demonstrated a higher apoptosis fraction in hypoxic KD cells with 100 μM PA than in SC cells (Supplementary Fig. S2). Furthermore, FAO activity without PA treatment was significantly suppressed in SC cells of both HCC cell lines under DMOG+ conditions than DMOG-, while FAO activity was remarkably elevated in KD cells (Fig. 3D). PA treatment further elevated FAO activity in KD cells of both HCC cell lines under DMOG+, as well as DMOG-, conditions (Fig. 3D). These results suggested that hypoxia could suppress FAO activity in HCC cells, but this was disrupted by HIF-1a KD. Next, we evaluated the effect of HIF-1α KD on ROS generation in HCC cells with PA treatment. As shown in Fig. 3E, intracellular ROS production was more strongly increased in hypoxic KD cells with PA treatment in a dose-dependent manner compared with in hypoxic SC cells. In addition, we analyzed the effect of HIF-1α KD on triglyceride (TG) generation with or without PA treatment. In this study, TG generation was microscopically evaluated by fat deposition, which was evaluated by the oil red staining intensity (Fig. 3F, G). In SC cells with no treatment, oil red staining in the cytoplasm was increased under hypoxia, and the intensity was further enhanced by 50 μM PA treatment (Fig. 3F). However, much weaker staining was observed in 50 μM PA-treated KD cells under hypoxia compared with SC cells (Fig. 3F). As shown in Fig. 3G, hypoxia significantly elevated the oil red value in SC cells, even with no treatment, compared with normoxia. PA treatment further increased the oil red staining value in hypoxic SC cells. In contrast, hypoxia-induced elevation of the oil red value was not observed in KD cells with or without PA treatment (Fig. 3G).

### LC accelerated cell apoptosis in PA-treated HIF-1a KD cells under hypoxia

**Fig. 4:**
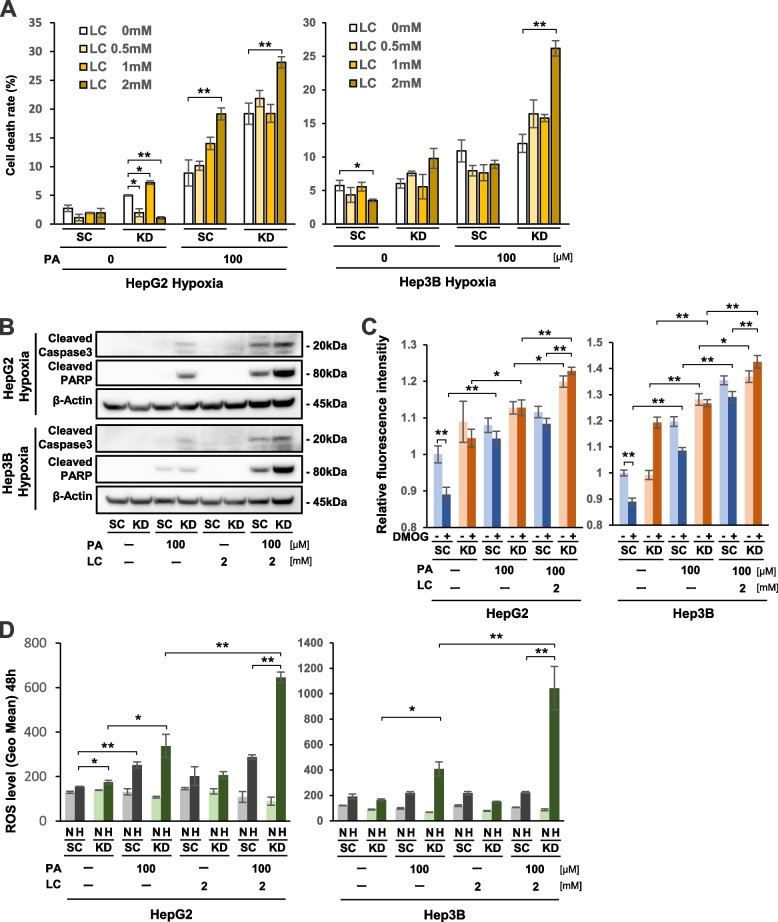
Synergistic effects of LC on lipo-apoptosis in HIF-1α knockdown (KD) cells with PA treatment under hypoxia. **A** Cell death rates in KD and scramble control (SC) cells of two HCC cell lines with a combination treatment of PA (0, 100 μM) with LC (0, 0.5 mM, 1 mM, 2 mM) under hypoxia for 48 hours. **B** Western blot analysis of cleaved caspase 3 and cleaved PARP protein expression in KD and SC cells that were treated with 100 μM PA and/or 2 mM LC under 48 hours hypoxia. **C** Fatty acid β-oxidation (FAO) activity in KD and SC cells under normoxia (DMOG-) and hypoxia mimicking (DMOG+) conditions with 100 μM PA and/or 2 mM LC for 12 hours. **D** Reactive oxygen species (ROS) levels in KD and SC cells with 100 μM PA and/or 2 mM LC treatment under normoxia (N) and hypoxia (H) for 48 hours. Values are presented as the mean ± standard error of the mean (SEM) (**A**,** C**,** D**). N.S.: not significant, *P < 0.05, **P < 0.01 versus control

We next investigated if an additional treatment with LC, which transfers FAs into mitochondria [40], could promote PA-induced lipo-apoptosis in HIF-1α KD cells under hypoxia. As shown in Fig. 4A, 2 mM LC treatment significantly increased cell death rates in 100 μM PA-treated KD cells under hypoxia in both HCC cell lines compared with treatment with PA alone. In contrast, neither PA nor PA + LC treatment increased cell death rate in HIF-1α KD cells under normoxia, compared with no treatment (Supplement Fig. S3). Western blot analysis showed that the expression levels of apoptosis-related proteins were elevated following the addition of LC in PA-treated KD cells under hypoxia in both cell lines (Fig. 4B). Flow cytometric analysis demonstrated a synergistic effect of LC treatment on PA induced-apoptosis in SC and KD cells from both HCC cell lines under hypoxia, with a higher apoptotic fraction observed in KD cells compared with SC cells (Supplement Fig. S4). The FAO levels were further elevated by LC addition in PA-treated KD cells under both DMOG- and DMOG+ conditions (Fig. 4C). There was also a remarkable elevation of ROS levels in hypoxic KD cells with the combination treatment of PA and LC compared with PA treatment alone (Fig. 4D). In summary, these findings suggested that the combination treatment of PA + LC more strongly activated the FAO pathway in hypoxic KD cells compared with PA alone. As a result, a larger amount of ROS might be produced and lead to a stronger induction of cell apoptosis in hypoxic KD cells.

### Regulation of gene expression related to fatty acid metabolism by HIF-1 under hypoxia

**Fig. 5:**
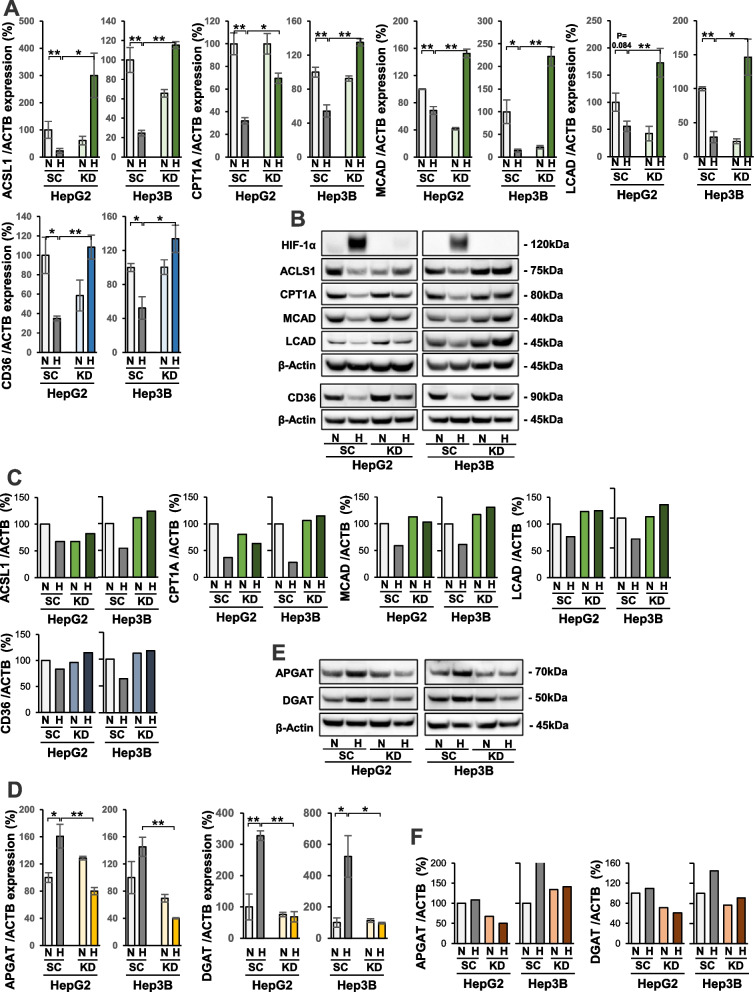
Regulation of fatty acid metabolism-related genes by HIF-1α under hypoxia. **A** qRT-PCR analysis of mRNA expression levels of genes encoding fatty acid β-oxidation (FAO)-related enzymes ACSL1, CPT1, MCAD, and LCAD, as well as fatty acid transporter CD36, in HIF-1α knockdown (KD) and scramble control (SC) HepG2 and Hep3B cells. **B** Western blot analysis of ACSL1, CPT1, MCAD, LCAD, and CD36 protein expression in KD and SC cells. **C** Quantification of Western blot analysis of ACSL1, CPT1, MCAD, LCAD, and CD36 protein expression. **D** qRT-PCR analysis of mRNA levels of triglyceride (TG) synthesis-related genes APGAT and DGAT in KD and SC cells. **E** Western blot analysis of APGAT and DGAT protein expression in KD and SC cells. **F** Quantification of Western blot analysis of APGAT, DGAT protein expression. Data are expressed as the mean ± standard error of the mean (SEM) and are representative of three independent experiments (**A**,** D**). N.S.: not significant, *P < 0.05, **P < 0.01 versus control

To further elucidate the critical role of HIF-1 in the reprogramming of fat metabolism in HCC cells under hypoxia, we analyzed the mRNA expression levels of genes encoding key enzymes related to fat metabolism using the HIF-1α KD and control SC HepG2 and Hep3B cells (Fig. 5). Results of qRT-PCR experiments showed that the mRNA expression levels of ACSL1, CPT1A, and MCAD were significantly decreased in both HepG2 and Hep3B SC cells under hypoxia compared with normoxia (Fig. 5A). In addition, LCAD mRNA expression levels were significantly lower under hypoxia than normoxia in Hep3B cells, whereas this trend was seen in HepG2 cells but was not statistically significant (*P* = 0.084). Nevertheless, hypoxic suppression of these genes was significantly reversed in KD cells under hypoxia compared with SC cells (Fig. 5A). Furthermore, the mRNA expression levels of CD36, the transporter involved in FA uptake [41], were significantly suppressed in SC cells under hypoxia in comparison with normoxia. Its expression was remarkably elevated in hypoxic KD cells (Fig. 5A). Western blot analysis also demonstrated lower protein expression levels of the FAO progressing enzymes and CD36 under hypoxia compared with normoxia in SC cells. However, this observed decreased expression was restored in hypoxic KD cells (Fig. 5B, C). In contrast, the mRNA expression levels of genes related to lipid droplets for storage, such as APGAT and DGAT, were elevated in SC cells under hypoxia compared with normoxia (Fig. 5D). This hypoxia-induced expression was significantly decreased in hypoxic KD cells compared with SC cells (Fig. 5D). Western blot analysis also confirmed that the hypoxia-induced expression of these enzymes in SC was not seen in hypoxic KD cells (Fig. 5E, F).

Taken together, these analyses using HIF-1α KD cells and control SC cells suggested that the expression patterns of the FAO progressing enzymes ACSL1, CPT1A, MCAD, and LCAD, as well as of FA transporter CD36, were negatively regulated by HIF-1 in hypoxic HCC cells. However, the lipid storage promoting enzymes APGAT and DGAT were positively regulated by HIF-1 (Table 1).

**Table 1:** Regulation of fatty acid metabolism-related gene expression in hepatocellular carcinoma (HCC) cells under hypoxic conditions

gene	Function in FA metabolism	HepG2		Hep3B	
		mRNA expression in Hypoxia	Regulation factor	mRNA expression in Hypoxia	Regulation factor
ACSL1	FAO	down	HIF-1	down	HIF-1
CPT1A		down	HIF-1	down	HIF-1
ACADM		down	HIF-1	down	HIF-1
ACADL		down	HIF-1	down	HIF-1
CD36	uptake of FA	down	HIF-1	down	HIF-1
AGPAT	Lipid droplets	up	HIF-1	up	HIF-1
DGAT		up	HIF-1	up	HIF-1

### HIF-1α inhibitor YC-1 increased FAO activity and enhanced lipo-apoptosis by PA plus LC treatment in HepG2 cells under hypoxia

**Fig. 6:**
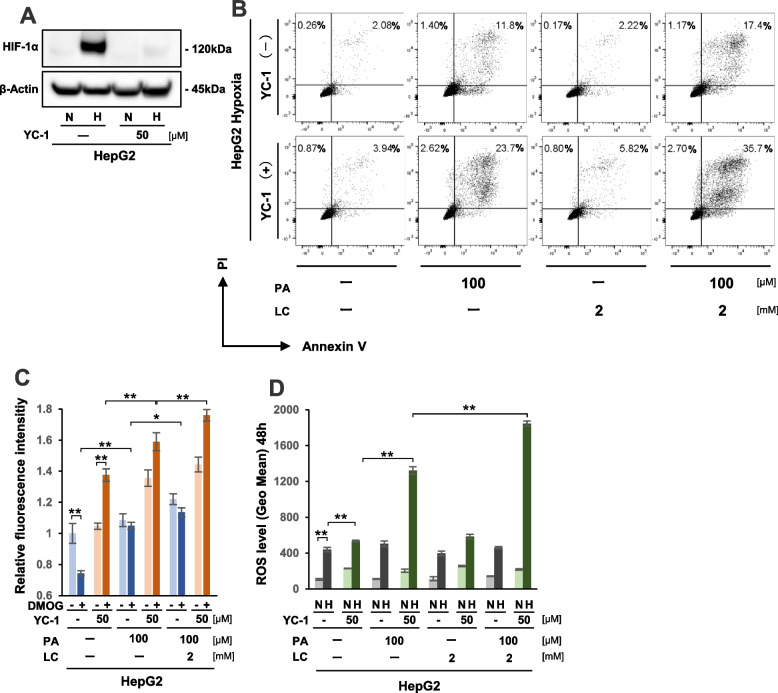
YC-1 combined with PA + LC treatment effectively increased lipo-apoptosis in HepG2 cells under hypoxia. **A** Western blot analysis of HIF-1α protein expression in HepG2 cells with or without 50 μM YC-1 treatment under normoxia (N) and hypoxia (H) for 12 hours. **B** Analysis of apoptosis by flow cytometry with Annexin V and PI staining in HepG2 cells that were treated with or without YC-1, PA, and LC for 48 hours under hypoxia. Proportions of apoptotic cells are indicated by percentage of Annexin V−/PI+ or Annexin V+/PI+. **C** Analysis of in vitro fatty acid β-oxidation (FAO) activity in HepG2 cells that were treated with or without YC-1, PA, and LC for 12 hours under normoxia (DMOG-) and hypoxia mimicking (DMOG+) conditions. **D** Reactive oxygen species (ROS) levels in HepG2 cells that were treated with or without YC-1, PA, and LC under normoxia (N) and hypoxia (H) for 48 hours. Values are presented as the mean ± standard error of the mean (SEM) (**C**,** D**). N.S.: not significant, *P < 0.05, **P < 0.01 versus control

We next investigated if HIF-1 inhibitor YC-1 could effectively inhibit HIF-1 expression in HepG2 cells under hypoxia, as observed in HIF-1α KD cells. Western blot analysis confirmed that 50 μM YC-1 treatment strongly inhibited HIF-1α protein expression in hypoxic HepG2 cells (Fig. 6A). Flow cytometric analysis further showed that triple treatment composed of YC-1 with PA + LC most strongly elevated the apoptotic fraction of cells at 38.4% (2.7% + 35.7%) among all treatments examined in hypoxic HepG2 cells (Fig. 6B). We next analyzed the biological effect of YC-1 on FAO activity under normoxia (DMOG-) and mimicked hypoxia (DMOG+) (Fig. 6C). FAO activity in DMOG+ conditions was clearly elevated by YC-1 treatment (Fig. 6C). In particular, FAO activity was the highest in the HepG2 cells treated by YC-1 with PA + LC under DMOG+ conditions (Fig. 6C). In addition, ROS levels were most strongly elevated by YC-1 with PA + LC in hypoxic HepG2 cells (Fig. 6D). Collectively, these results showed that HIF-1 inhibitor YC-1 was an effective drug for inhibiting HIF-1α expression. Furthermore, we demonstrated that a combination therapy composed of YC-1 with PA + LC could strongly induce apoptosis in hypoxic HepG2 cells from excessive ROS generation via the FAO pathway.

### A combination treatment of PA + LC could suppress the growth of HIF-1α KD tumors in nude mice

**Fig. 7:**
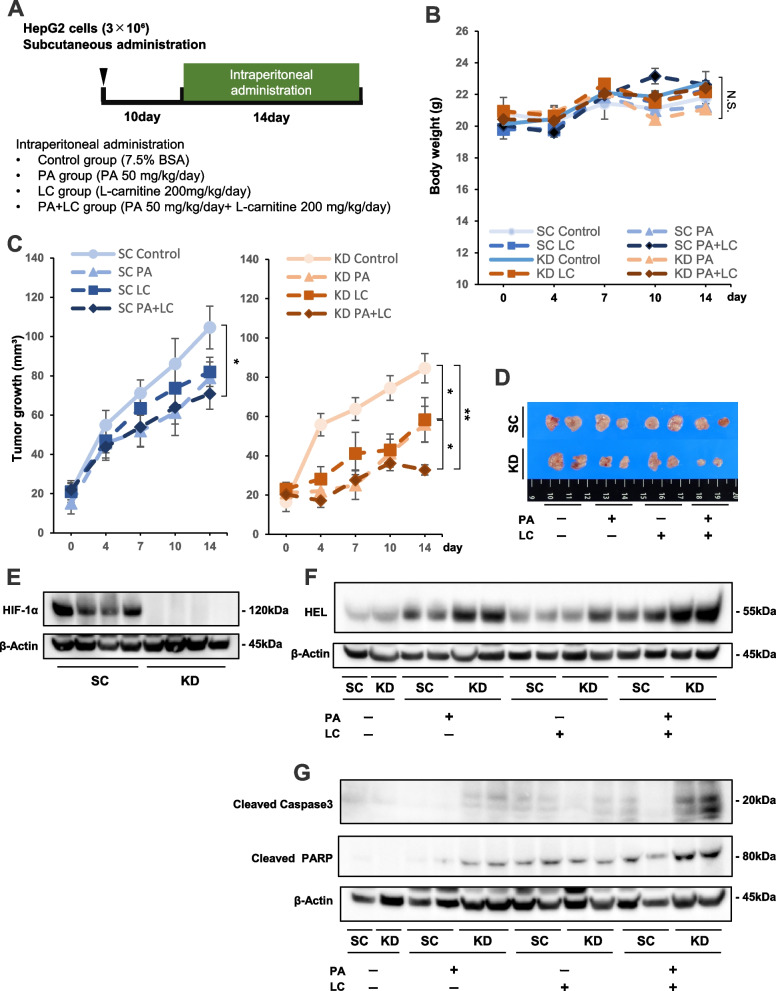
The in vivo effect of PA and LC on HIF-1α knockdown (KD) cell tumor xenografts. **A** Experimental schedule of HepG2 cell KD and scramble control (SC) tumors in nude mice that underwent intraperitoneal (i.p.) administration of drugs. The four treatment groups included control, PA alone, LC alone, and PA + LC. **B** Body weight transition of mice during the treatment period. **C** Transition of mean volume of KD and SC tumors that underwent four drug treatments (control, PA alone, LC alone, and PA + LC). **D** Macroscopic appearance of KD and SC tumor xenografts from the four treatment groups. **E** Western blot analysis of HIF-1α protein expression in KD and SC xenograft tumors from the four treatment groups. **F** Western blot analysis of hexanoyl lysine (HEL) protein expression in KD and SC xenograft tumors from the four treatment groups. Tumors were randomly selected from each group and subjected to this analysis. **G** Western blot analysis of cleaved caspase 3 and cleaved PARP protein expression in HIF-1α KD and SC xenograft tumors from the four treatment groups. Tumors were randomly selected from each group and subjected to this analysis. Values are presented as the mean ± standard error of the mean (SEM) (**B**,** C**). N.S.: not significant, *P < 0.05, **P < 0.01

We then investigated the in vivo effects of HIF-1α inhibition combined with PA + LC treatment on tumor xenograft growth. As shown in the experimental schedule (Fig. 7A), the HepG2 HIF-1α KD and SC cells were subcutaneously injected into nude mice. Daily intraperitoneal (i.p.) administration of drugs was started on day 10. The mice were sorted into four groups, including the control, PA alone, LC alone, and PA + LC groups. Drug administration was performed and continued for 14 days (Fig. 7A). Figure 7B shows the body weight transition of mice during the treatment period, with no significant difference in body weight observed among the four treatment groups. The tumor volume of SC and KD cells with each treatment was estimated daily for 14 days (Fig. 7C). In SC tumors, the mean tumor volume was significantly reduced by the combination treatment of PA + LC for 14 days compared with the control group (Fig. 7C). However, tumor volume was also significantly reduced in KD tumors even with treatment with PA or LC individually (Fig. 7C). Moreover, the strongest reduction in tumor volume was observed in KD tumors with the combination of PA + LC (Fig. 7C). Fourteen days after drug administration was initiated, the subcutaneous tumors were excised. As shown in Fig. 7D, the smallest tumor was macroscopically observed in KD tumors with PA + LC treatment among other tumors. Western blot analysis of the excised tumors showed HIF-1α protein expression in the SC tumors, but not in the KD tumors (Fig. 7E). The results indicated that the hypoxic region natively exists in the subcutaneous SC tumors. To evaluate ROS production in the excised tumors, expression of hexanoyl lysine (HEL), a marker molecule for fat peroxidation by ROS, was investigated. As shown in Fig. 7F, among all SC and KD tumor treatment groups, HEL was most strongly expressed in the KD tumors with PA + LC treatment. Additionally, this group also displayed the highest expression levels of cleaved caspase 3 and cleaved PARP (Fig. 7G).

### YC-1 combined with PA + LC treatment exhibited tumor suppression in vivo

**Fig. 8:**
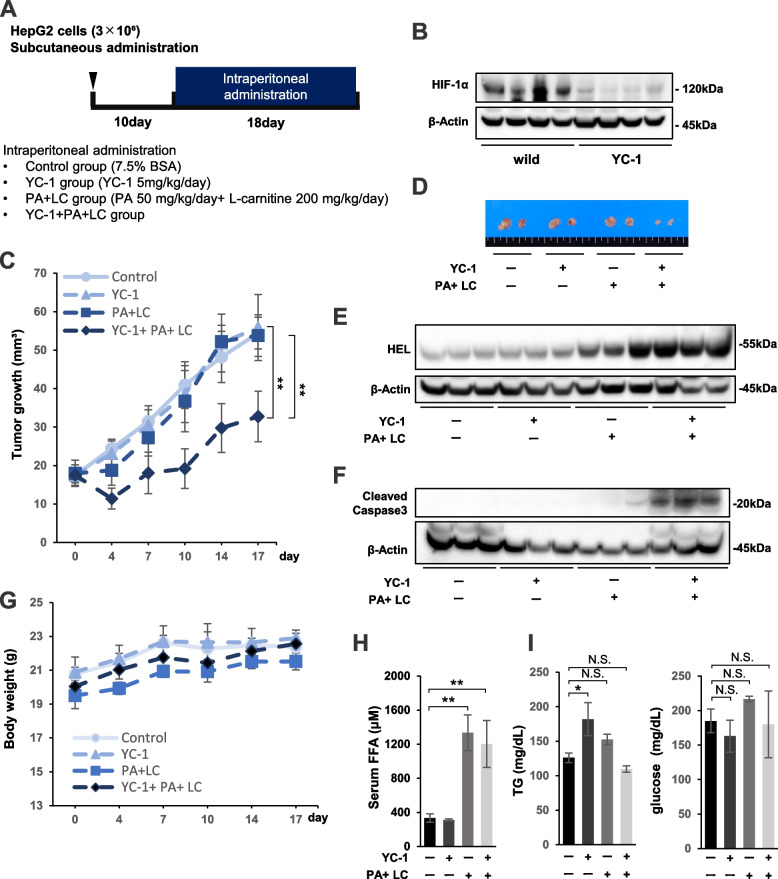
*The* in vivo effect of YC-1 combined with PA + LC treatment on tumor xenografts. **A** Experimental schedule of HepG2 xenograft tumors in the four treatment groups, including control, YC-1, PA + LC, and YC-1 + PA + LC. **B** Western blot analysis of HIF-1α protein expression in control and YC-1-treated xenograft tumors. Four tumors were randomly selected from each treatment group. **C** Mean tumor volume of HepG2 xenografts from the four treatment groups. **D** Macroscopic appearance of tumors from the four treatment groups. **E** Western blot analysis of hexanoyl lysine (HEL) protein expression in HepG2 xenograft tumors from the four treatment groups. Three tumors were randomly selected from each treatment group. **F** Western blot analysis of cleaved caspase 3 protein expression in HepG2 xenograft tumors from the four treatment groups. Three tumors were randomly selected from each treatment group. **G** Body weight transition of mice during the treatment period. **H** Blood examination of serum free fatty acid (FFA) levels in mice from the four treatment groups. **I** Blood examination of serum triglyceride (TG) and glucose levels in mice from the four treatment groups. Values are presented as the mean ± standard error of the mean (SEM). N.S.: not significant, *P < 0.05, **P < 0.01 versus control

Lastly, we assessed the in vivo effects of HIF-1 inhibitor YC-1 with or without PA + LC on HepG2 cell tumor growth. According to the experimental schedule, four groups of mice were examined, including the control, YC-1 alone, PA + LC, and YC-1 with PA + LC groups. These drugs were i.p. administered daily to tumor-bearing mice (Fig. 8A). We first confirmed that administration of YC-1 at 5 mg/kg strongly inhibited HIF-1α expression in HepG2 tumors (Fig. 8B). YC-1 combined with PA + LC treatment most strongly inhibited tumor growth among all groups (Fig. 8C). The smallest excised tumor was observed in the YC-1 with PA + LC treatment group (Fig. 8D). Furthermore, western blot analysis revealed that the highest HEL protein expression levels were observed in the tumors that were treated with YC-1 and PA + LC (Fig. 8E). This group also demonstrated the strongest expression of cleaved caspase 3 (Fig. 8F). Additionally, the body weight of the mice did not show any significant difference among each treatment group (Fig. 8G). Blood examination suggested that FFA serum levels were significantly higher in mice treated with PA + LC and YC-1 with PA + LC compared with the others (Fig. 8H). However, a significantly higher TG concentration was not observed in the PA + LC or YC-1 with PA + LC group (Fig. 8I). Moreover, fat deposition in the liver, which was evaluated by oil red staining, was not increased by the PA + LC or YC-1 with PA + LC treatment (Supplementary Fig. S5). In addition, there was no difference in serum glucose levels among the four groups (Fig. 8I).

## Discussion

In the present study, we first demonstrated that PA treatment at 100 to 200 μM could significantly induce lipo-apoptosis in HepG2 and Hep3B cells in a hypoxia-dependent manner. PA also increased ROS production in the hypoxic HCC cells. Moreover, antioxidant agent NAC treatment reduced both the PA-induced apoptosis and ROS generation, suggesting the PA-induced lipo-apoptosis was caused by excessive ROS production under hypoxia. Therfore, we hypothesized that hypoxia dependent lipo-apoptosis by PA treatment could possibly be an effective anti-HCC therapy because cancer cells in HCC tumors grow in a hypoxic environment, while normal cells do not. Therefore, we further aimed to clarify the mechanism by which lethal ROS are generated by PA-treated HCC cells under hypoxia. We performed in vitro assays to analyze FAO activity in HCC cell lines under hypoxia-mimicking conditions using a PHD inhibitor DMOG [42]. In HepG2 and Hep3B cells, FAO activity was remarkably suppressed under hypoxia (DMOG+) compared with normoxia (DMOG-), indicating that HIF-1α might suppress FAO activity in hypoxic HCC cells. Moreover, we showed that additional PA treatment from 50 to 100 μM could increase FAO activity in a dose-dependent manner, even under mimicked hypoxia conditions. From this, we hypothesized that PA-induced lipo-apoptosis might be triggered via forced activation of the FAO pathway from overloaded PA under hypoxia, in which acetyl-CoA was overproduced and entered the TCA cycle, leading to excess ROS generation in OXPHOS through ETC. To prove this hypothesis, we further examined if siRNA-mediated knockdown of CPT1A could inhibit FAO activity and reduce ROS production, resulting in attenuated apoptosis in hypoxic HCC cells. As expected, CPT1A KD significantly reduced FAO activity in two HCC cell lines under normoxia (DMOG-) and hypoxia mimicking (DMOG+) conditions. Moreover, hypoxia-induced ROS generation was decreased in CPT1A KD cells with PA treatment, which led to reduced apoptosis rates. These results provided evidence that PA-induced apoptosis originated from the forced activation of the FAO pathway by PA overload in hypoxic HCC cells.

Previously, a noteworthy report demonstrated that HIF-1α can inhibit FAO. This was specifically through HIF-1α-mediated suppression of PGC-1β, which inhibits expression of FAO enzymes LCAD and MCAD in hypoxic Hep3B cells [43]. Another report conversely showed that hypoxia causes TG accumulation by HIF-1α-mediated stimulation of lipin 1 expression in Huh7 HCC cells, whereby hypoxia-induced lipin 1 can accelerate conversion of phosphatidic acid to diacylglycerol (DAG) in TG synthesis [44]. In the present study, we clearly showed that apoptosis from 100 μM PA treatment was significantly enhanced in KD cells under hypoxia compared with SC cells. Moreover, both FAO activity and ROS generation were remarkably elevated in HIF-1α KD cells under hypoxia. In sharp contrast, TG generation, which was assessed by oil red staining, was inhibited by HIF-1α KD under hypoxic conditions. Taken together, these results indicated that HIF-1α KD increased PA-induced lipo-apoptosis in HCC cells under hypoxia. Suppression of FAO and promotion of TG generation were prevented by HIF-1α KD under hypoxia.

LC is an essential compound for transferring long chain FAs to mitochondria and functions as a so-called “carnitine shuttle” [40]. Therefore, we expected that LC treatment would further elevate FAO activity and enhance PA-induced lipo-apoptosis in hypoxic KD HCC cell lines. A series of cell death assays revealed that combining 2 mM LC and 100 μM PA synergistically increased cell apoptosis rates in hypoxic KD cells of two HCC cell lines. The results strongly indicated that LC addition might increase PA transfer into the mitochondria matrix and promote FAO, resulting in further ROS generation through OXPHOS in hypoxic KD cells. As described above, this study indicated that HIF-1α possibly plays central roles in suppressing FAO, as well as in promoting TG accumulation, to prevent PA-induced apoptosis in hypoxic HCC cells. Conversely, we also demonstrated that HIF-1α KD is a critical effector for elevating the susceptibility of HCC cells to apoptosis with PA treatment under hypoxia.

To verify the essential mechanism of HIF-1α in hypoxia-induced reprogramming of fat metabolism, expression of genes related to FAO, TG accumulation, and FA uptake were analyzed in HIF-1α KD and SC cells of the two HCC cell lines. For FAO in the mitochondria, FAs are first converted to ACS [45, 46], then CPT1A converts acyl-CoA to acylcarnitine [43, 45]. Acylcarnitine in the matrix is converted to acyl-CoA by CPT2, which is then finally converted to acethy-CoA by MCAD and LCAD [47]. In this study, we analyzed the expression levels of FAO-related genes ACSL1, CPT1A, MCAD, and LCAD, finding that these four enzymes were suppressed in control SC cells under hypoxia, while their expression patterns were reversed by HIF-1α KD. Similarly, expression of CD36, one of the major transporters for FA uptake, is negatively regulated by HIF-1α. Additionally, we demonstrated that fat deposition was increased by hypoxia and further promoted by PA treatment in SC cells, whereas the deposition was reduced in HIF-1α KD cells. During the process of fat deposition, esterification-related enzymes, including DGAT and APGAT, play a key role in converting FAs to TGs [48, 49]. In this study, we showed that hypoxia could increase the expression levels of both DGAT and APGAT in SC cells, while the hypoxia-induced expression was attenuated in KD cells. These data indicated that HIF-1α could promote DGAT and APGAT mRNA upregulation in hypoxic HCC cells. Taken together, gene expression analysis revealed the mechanism by which HIF-1α can prevent ROS synthesis in OXPHOS to avoid PA-induced apoptosis in hypoxic HCC cells. HIF-1α can suppress the mRNA expression of FAO-related genes and CD36, as well as conversely support the upregulation of genes related to TG generation.

YC-1 is a drug that was developed for circulatory disorders by specifically inhibiting platelet aggregation and vascular contraction. It is also known as a HIF-1α inhibitor [50]. For future applications of this study’s findings to clinical trials, we aimed to substitute YC-1 treatment with specific siRNA-mediated knockdown for inhibition of HIF-1α expression in hypoxic HCC cells. Western blot analysis showed that 50 μM YC-1 treatment could completely inhibit hypoxia-induced HIF-1α expression in HepG2 cells. Additionally, this treatment did not show cytotoxicity under normoxia, indicating that YC-1 at 50 μM might play a major role in inhibiting HIF-1a expression. In fact, YC-1 treatment strongly increased FAO activity, ROS production, and apoptosis rates in HepG2 cells with PA + LC treatment under hypoxia.

To establish an in vivo model of YC-1 with PA + LC therapy for HCC, we aimed to evaluate the anti-tumor effects of this treatment approach on xenograft tumor growth in mice. Prior to YC-1 with PA + LC treatment, the anti-tumor effects of PA + LC was assessed in HIF-1α KD and SC HepG2 cell tumors. We first observed HIF-1α expression in HepG2-SC tumors, thus confirming the hypoxic region that spontaneously arose in these xenograft tumors. In HIF-1α KD tumors, i.p. administration of PA + LC remarkably inhibited tumor growth and induced the strongest levels of apoptosis and fat peroxidation in KD tumors among all treatments analyzed. These results indicated that lipo-apoptosis was possibly induced in the hypoxic region within the PA + LC-treated KD tumors via excess ROS production. Lastly, we performed an in vivo study of YC-1 combined with PA + LC treatment for HCC in nude mice. Previous studies reported in vivo tumor suppression with YC-1 monotherapy at doses ranging from 30 to 100 mg/kg/day [51–54]. With this model, we first showed that YC-1 at 5 mg/kg/day showed a strong inhibitory effect on HIF-1a expression levels in tumor xenografts. A triple combination of YC-1 with PA + LC exhibited remarkable growth inhibition and apoptosis in HepG2 tumors compared with the control treatment. However, treatment with YC-1 alone or the PA + LC combination did not show significant tumor inhibitory effects. Serum concentrations of FFA in the mice were significantly increased with PA + LC or YC-1 and PA + LC treatment compared with the others. However, oil red staining did not show any elevated fat deposition in the liver compared with the control, indicating that PA treatment at 50 mg/kg/day did not result in hepatocellular fat storage in this in vivo study (Supplementary Fig. S4). In addition, TG and BS serum concentrations in the mice were not affected by YC-1 with PA + LC treatment compared with the control. Therefore, we propose a novel pre-clinical therapy composed of YC-1 with PA + LC for HCC, in which lipo-apoptosis is selectively induced within the hypoxic region of HCC tumors. Moreover, this combination therapy did not show any significant toxicity in the mice.

## Conclusions

**Fig. 9:**
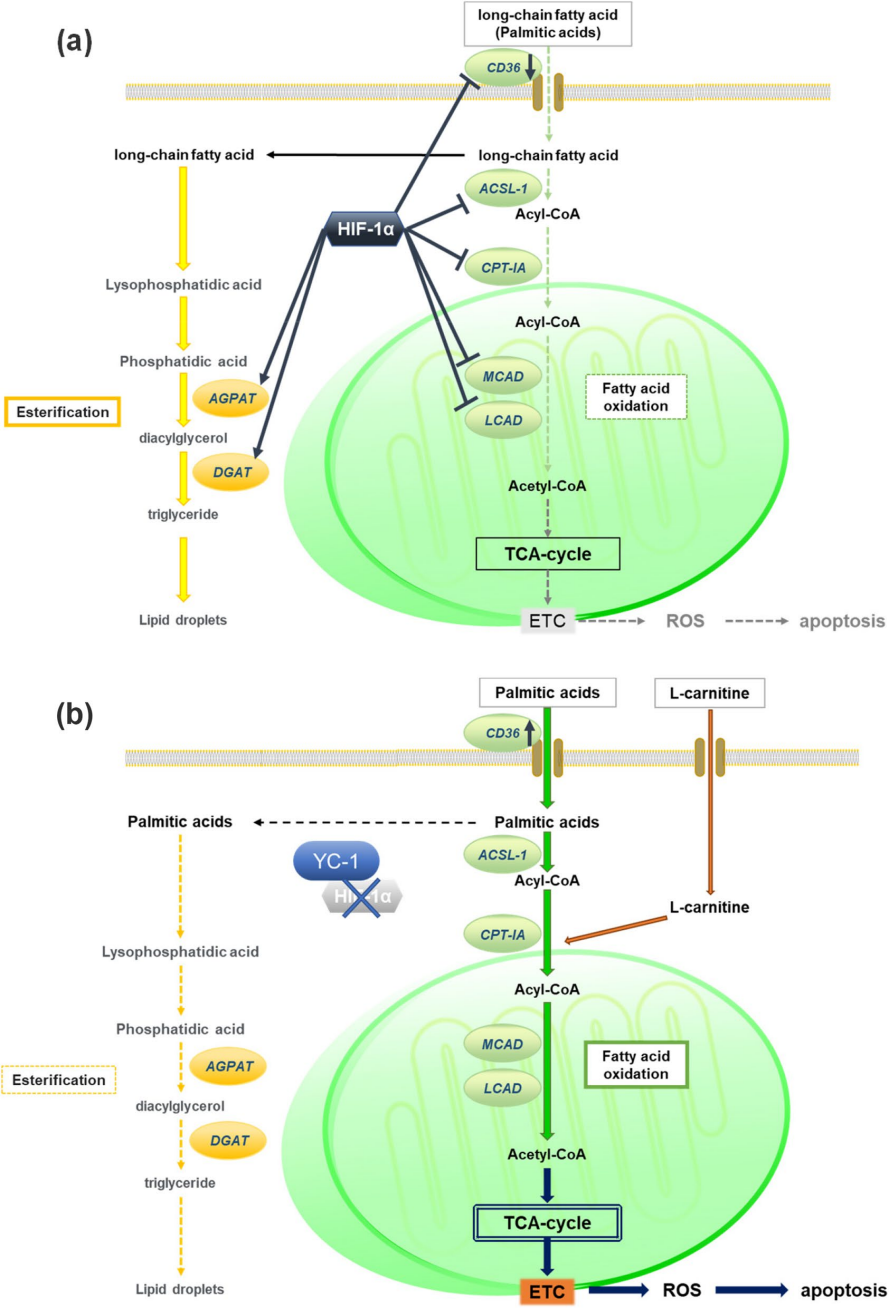
Schematic representation of YC-1 with PA + LC treatment to target hypoxic within HCC tumors. **A** Reprograming of fatty acid metabolism by HIF-1α in hypoxic regions within HCC tumors. HIF-1α inhibits gene expression of CD36, ACSL-1, CPT-1A, MCAD, and LCAD, leading to suppressed fatty acid β-oxidation (FAO) activity in hypoxic HCC cells. In contrast, HIF-1α induces expression of AGPAT and DGAT to promote triglyceride (TG) synthesis and increases lipid droplets in hypoxic HCC cells. **B** The lipo-apoptotic effect of YC-1 with PA + LC treatment on hypoxic HCC cells. Inhibition of HIF-1α expression by YC-1 increases fatty acid uptake and elevates FAO activity in hypoxic HCC cells by restoring expression of CD36 and FAO-related enzymes, respectively. Additionally, inhibited HIF-1α expression by YC-1 suppresses hypoxic upregulation of AGPAT and DGAT, leading to reduced TG synthesis. Taken together, YC-1 treatment causes forced activation of FAO and leads to lethal reactive oxygen species (ROS) production through TCA cycle to ETC axis in hypoxic HCC cells. Moreover, additional treatment with PA plus LC further promotes FAO activity, increases ROS production, and synergistically enhances cell apoptosis

In summary, our proposed in vitro apoptotic mechanism of YC-1 with PA + LC therapy in hypoxic HCC cells is illustrated in Fig. 9. This study presents a novel therapeutic strategy in which YC-1 with PA + LC therapy targets the hypoxic cancer cells within HCC tumors. Hypoxia-induced reprogramming of fat metabolism is destroyed, with the HIF-1α-mediated shift from FAO to lipid storage being inhibited by YC-1. FAO is thereby forcedly activated, with the activity further enhanced by an overload of PA + LC. As a result, excess ROS are produced during OXPHOS and apoptosis is specifically induced in HIF-1α-deficient HCC cells. Additionally, this treatment may also supply FAs to FAO in normal cells that are within a normoxic environment, where apoptosis is not induced. Therefore, YC-1 with PA + LC may be a promising anti-cancer therapy because hypoxic HCC cells with malignant behaviors may be selectively killed, while normal cells living in normoxia can survive and receive energy from the PA + LC treatment.

### Supplementary Information

**Additional file 1: Supplementary Fig. S1.** Flow cytometric analysis of apoptosis with double staining of Annexin V and PI. HepG2 and Hep3B cells were treated with PA at 0, 50, 100, and 200 μM concentrations under hypoxia for 48 hours. Hepatocellular carcinoma (HCC) cells not treated with PA under normoxia were also analyzed as a control. Detection of Annexin V−/PI+ or Annexin V+/PI+ was considered as apoptotic cells. The population was indicated by percentage.

**Additional file 2: Supplementary Fig. S2.** Analysis of apoptosis by flow cytometry with Annexin V and PI staining. KD and SC cells were treated with PA (0–100 μM) under hypoxia for 48 hours. Untreated HCC cells under normoxia were also analyzed as a control. Detection of Annexin V−/PI+ or Annexin V+/PI+ was assessed as apoptotic cells, and the population is indicated by percentage.

**Additional file 3: Supplementary Fig. S3.** Cell death rates in KD and scramble control (SC) cells of two HCC cell lines with a combination treatment of PA (0, 100 μM) with LC (0, 0.5 mM, 1 mM, 2 mM) under Normoxia for 48 hours.

**Additional file 4: Supplementary Fig. S4.** Analysis of apoptosis by flow cytometry with Annexin V and PI staining in HIF-1α KD and SC cells that were treated with 100 μM PA and/or 2 mM LC under hypoxia for 48 hours. Percentage of apoptotic fraction is shown by percentage (Annexin V−/PI+ or Annexin V+/PI+).

**Additional file 5: Supplementary Fig. S5.** Oil red staining of liver tissues was performed, and the representative images are shown from the four groups (control, YC-1 alone, PA + LC, YC-1 plus PA + LC).

## Data Availability

The dataset generated and/or analyzed during the current study are available from the corresponding author upon reasonable request.

## Abbreviations

YC-1: 3-(5′-hydroxymethyl-2′-furyl)-1-benzylindazole
CPT1A: carnitine palmitoyltransferase1A
ACSL1: Acyl-CoA Synthetase Long Chain Family Member 1
MCAD: Acyl-CoA Dehydrogenase Medium Chain
LCAD: Acyl-CoA Dehydrogenase long Chain
DGAT1: diacylglycerol O-acyltransferase 1
APGAT1: 1-acylglycerol-3-phosphate O-acyltransferase 1
DAG: diacylglycerol
TG: triglyceride
ACS: acyl-CoA by acyl-coenzyme A synthases
