# Supplementary figures and images for: A HIF-1α inhibitor combined with palmitic acid and L-carnitine treatment can prevent the fat metabolic reprogramming under hypoxia and induce apoptosis in hepatocellular carcinoma cells
Source: Cancer Metab. 2023 Dec 8;11:25. doi: 10.1186/s40170-023-00328-w (PMC10709876; doi:10.1186/s40170-023-00328-w)

## Slide 1
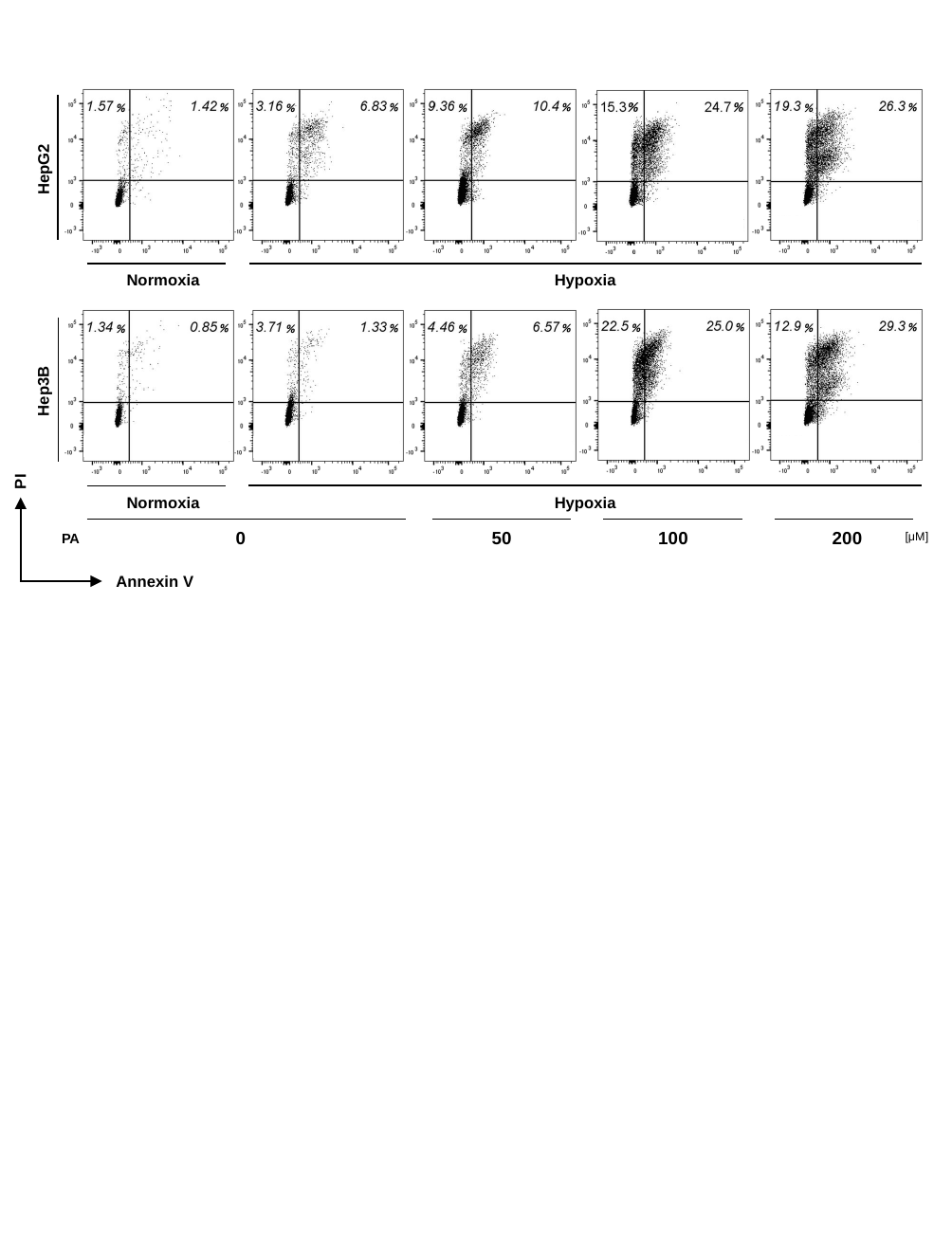

HepG2
Normoxia
Hypoxia
Hep3B
PI
Annexin V
Normoxia
Hypoxia
0
50
100
200
[μM]
PA

Supplement: Supplementary file 1 — Additional file 1: Supplementary Fig. S1. Flow cytometric analysis of apoptosis with double staining of Annexin V and PI. HepG2 and Hep3B cells were treated with PA at 0, 50, 100, and 200 μM concentrations under hypoxia for 48 hours. Hepatocellular carcinoma (HCC) cells not treated with PA under normoxia were also analyzed as a control. Detection of Annexin V−/PI+ or Annexin V+/PI+ was considered as apoptotic cells. The population was indicated by percentage. [file 40170_2023_328_MOESM1_ESM.pptx]

## Slide 1
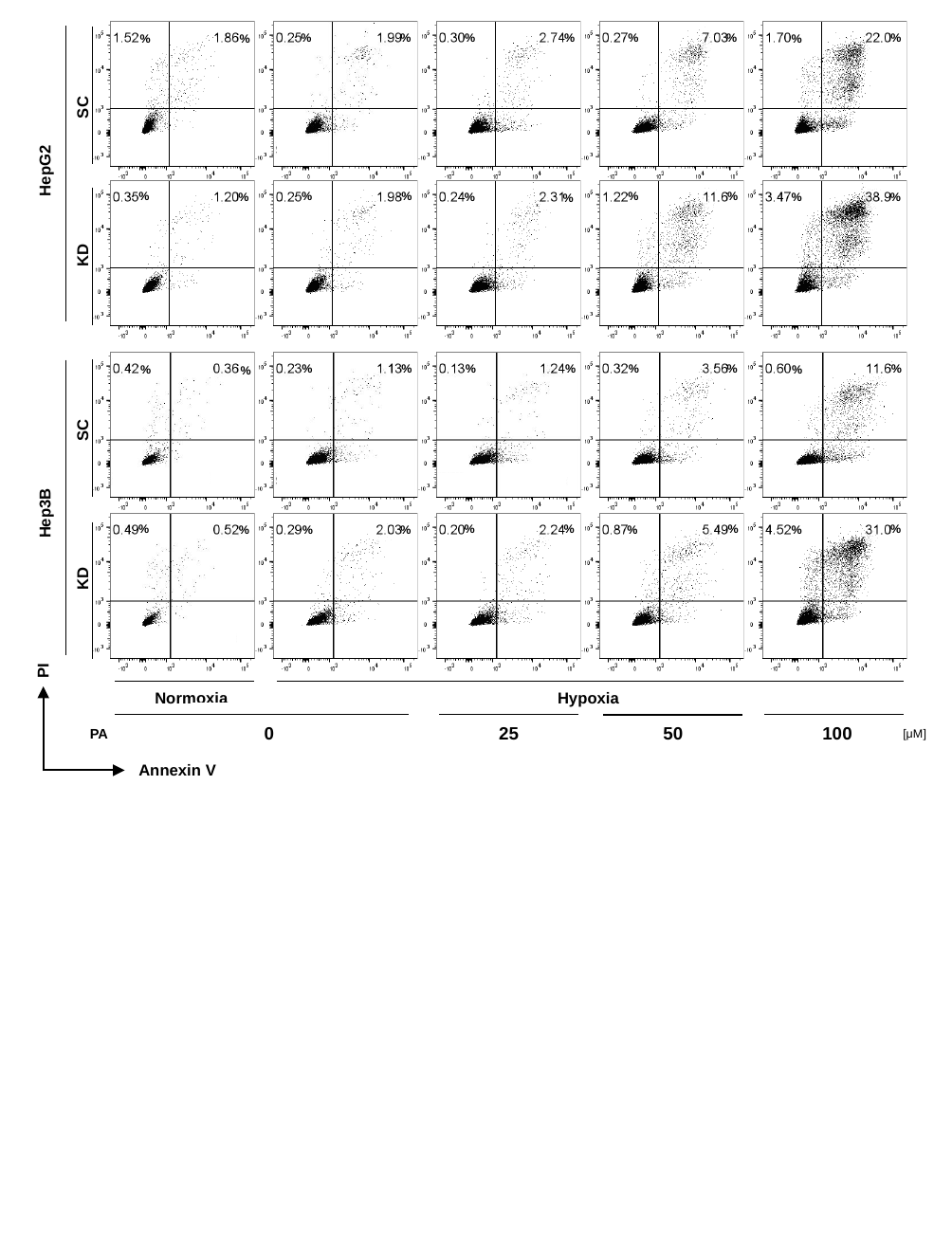

%
%
%
%
%
%
%
%
%
%
%
%
%
%
%
%
%
%
%
%
SC
HepG2
KD
%
%
%
%
%
%
%
%
%
%
%
%
%
%
%
%
%
%
%
%
SC
Hep3B
KD
PI
Annexin V
Normoxia
Hypoxia
0
25
50
100
PA
[μM]

Supplement: Supplementary file 2 — Additional file 2: Supplementary Fig. S2. Analysis of apoptosis by flow cytometry with Annexin V and PI staining. KD and SC cells were treated with PA (0–100 μM) under hypoxia for 48 hours. Untreated HCC cells under normoxia were also analyzed as a control. Detection of Annexin V−/PI+ or Annexin V+/PI+ was assessed as apoptotic cells, and the population is indicated by percentage. [file 40170_2023_328_MOESM2_ESM.pptx]

## Slide 1
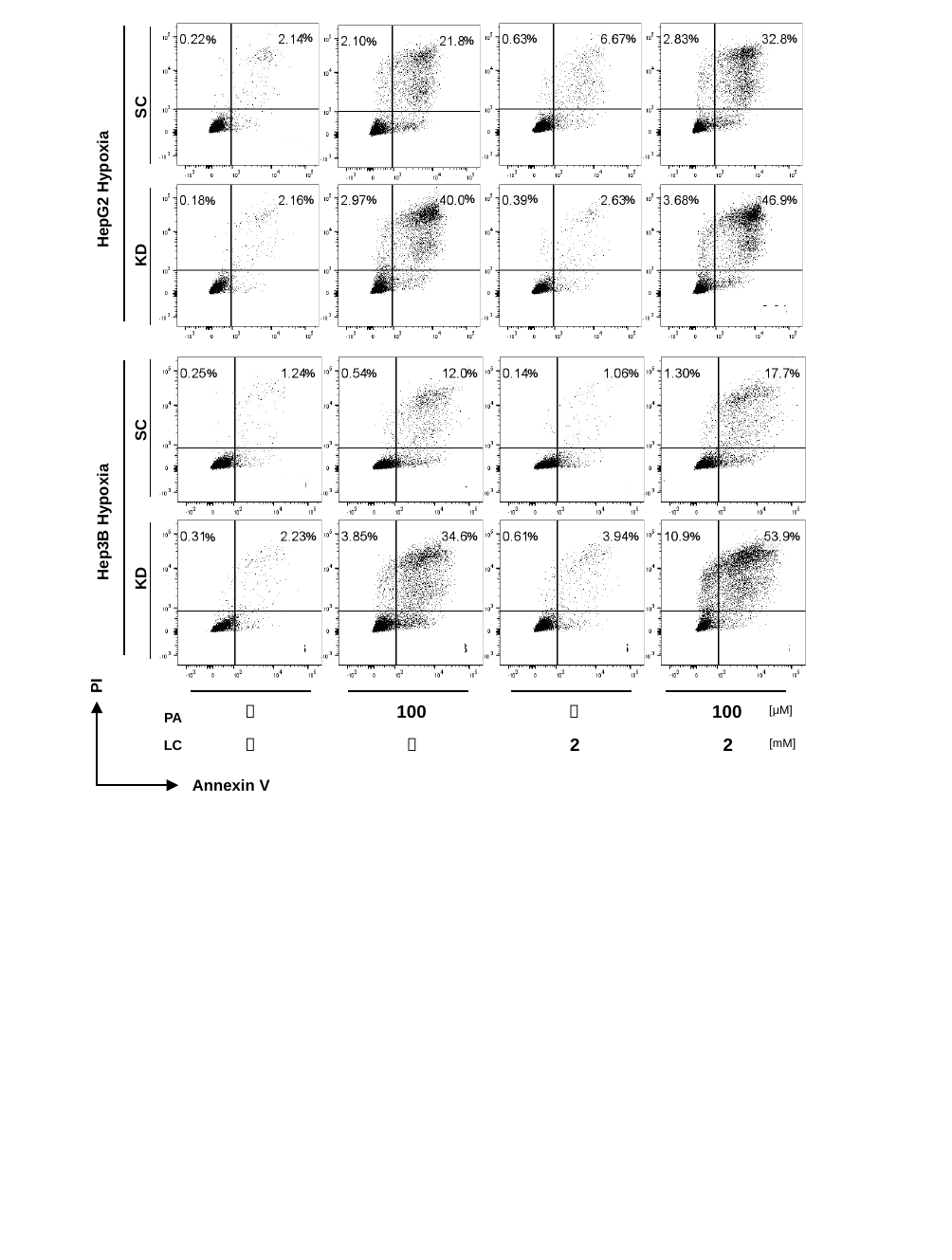

%
%
%
%
%
%
%
%
%
%
%
%
%
%
%
%
SC
HepG2 Hypoxia
KD
%
%
%
%
%
%
%
%
%
%
%
%
%
%
%
%
SC
Hep3B Hypoxia
KD
PI
Annexin V
ー
100
ー
100
[μM]
PA
ー
ー
2
2
[mM]
LC

Supplement: Supplementary file 4 — Additional file 4: Supplementary Fig. S4. Analysis of apoptosis by flow cytometry with Annexin V and PI staining in HIF-1α KD and SC cells that were treated with 100 μM PA and/or 2 mM LC under hypoxia for 48 hours. Percentage of apoptotic fraction is shown by percentage (Annexin V−/PI+ or Annexin V+/PI+). [file 40170_2023_328_MOESM4_ESM.pptx]

## Slide 1
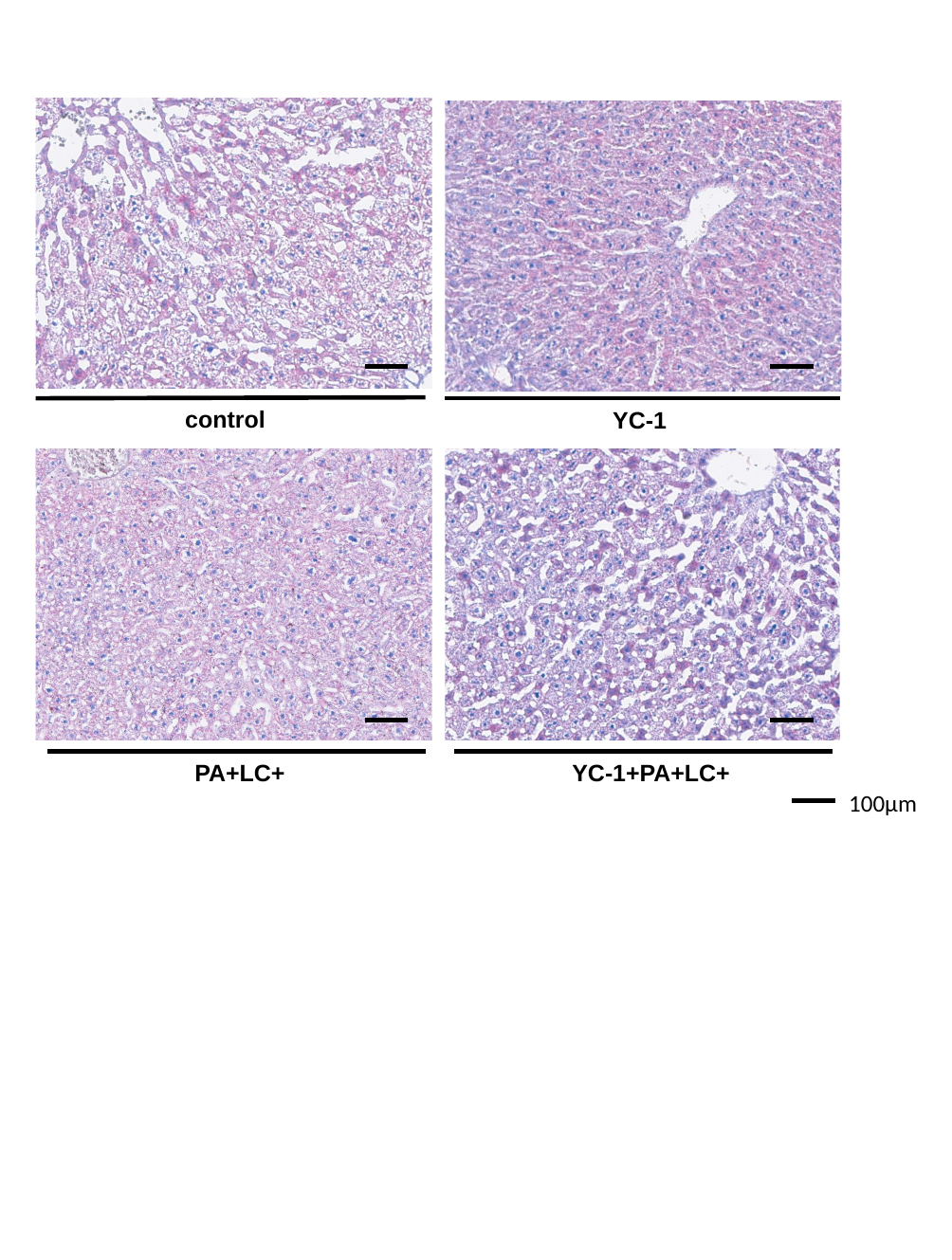

control
YC-1
YC-1+PA+LC+
PA+LC+
100μm

Supplement: Supplementary file 5 — Additional file 5: Supplementary Fig. S5. Oil red staining of liver tissues was performed, and the representative images are shown from the four groups (control, YC-1 alone, PA + LC, YC-1 plus PA + LC). [file 40170_2023_328_MOESM5_ESM.pptx]
