# Supplementary material for: A HIF-1α inhibitor combined with palmitic acid and L-carnitine treatment can prevent the fat metabolic reprogramming under hypoxia and induce apoptosis in hepatocellular carcinoma cells
Source: Cancer Metab. 2023 Dec 8;11:25. doi: 10.1186/s40170-023-00328-w (PMC10709876; doi:10.1186/s40170-023-00328-w)
Supplement: Supplementary file 3 — Additional file 3: Supplementary Fig. S3. Cell death rates in KD and scramble control (SC) cells of two HCC cell lines with a combination treatment of PA (0, 100 μM) with LC (0, 0.5 mM, 1 mM, 2 mM) under Normoxia for 48 hours. [file 40170_2023_328_MOESM3_ESM.pptx]

## Slide 1
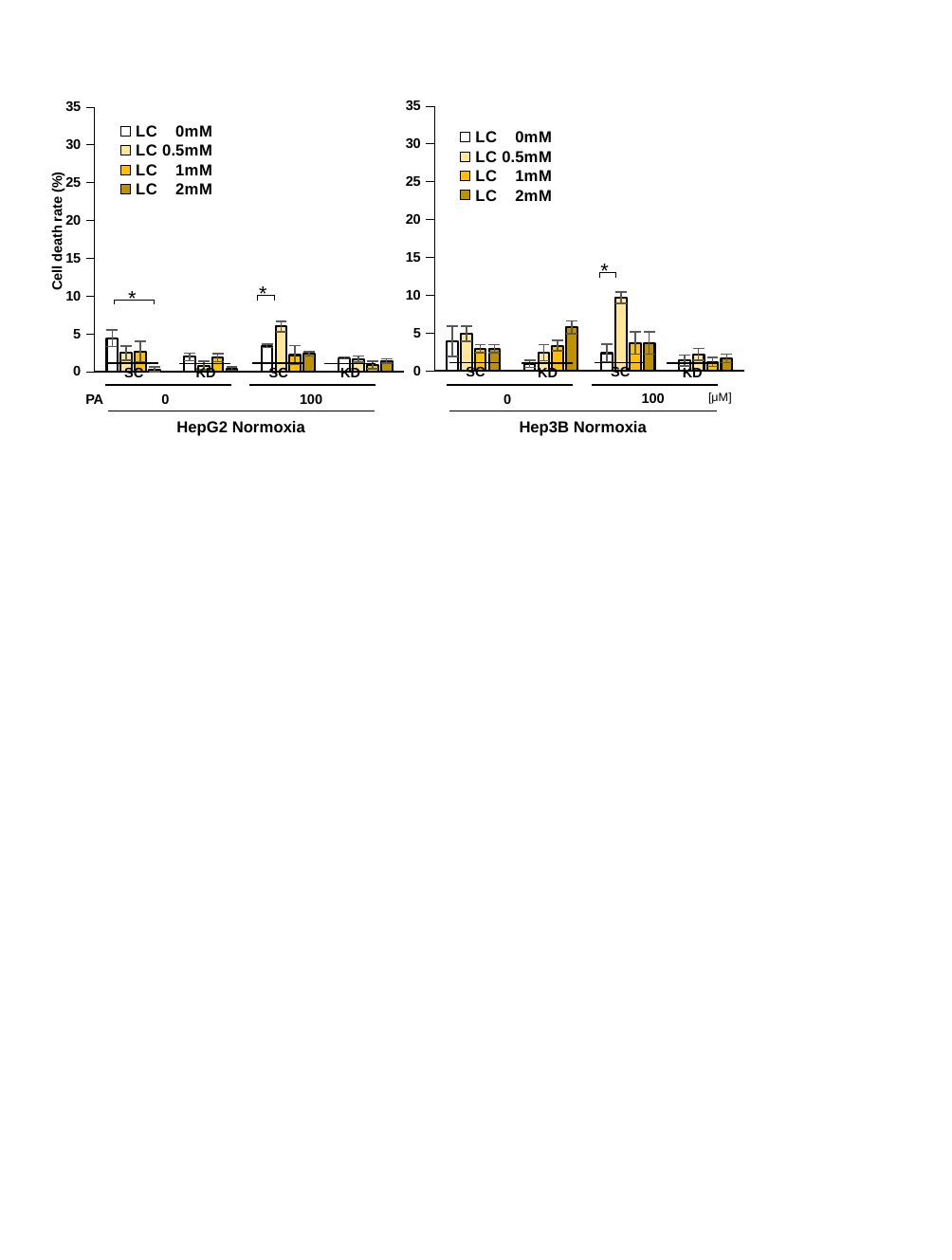

### Chart
| Category | LC 0mM | LC 0.5mM | LC 1mM | LC 2mM |
|---|---|---|---|---|
| 1 | 3.9025290345079213 | 4.88931849589478 | 2.9575623399084208 | 2.9575623399084208 |
| 2 | 0.9527335554732824 | 2.3927786765799133 | 3.3218177382169976 | 5.774050638947817 |
| 7 | 2.3454765646203413 | 9.668677533906646 | 3.693161837939712 | 3.693161837939712 |
| 8 | 1.3885082263950277 | 2.2081529994829387 | 1.1951719318195302 | 1.6577007884556043 |
### Chart
| Category | LC 0mM | LC 0.5mM | LC 1mM | LC 2mM |
|---|---|---|---|---|
| 1 | 4.443281200537797 | 2.4732981248781933 | 2.6534573471014085 | 0.32237266279819227 |
| 2 | 1.978998952067703 | 0.8104143747708102 | 1.8712912845520862 | 0.4404321307921985 |
| 7 | 3.4339142411903887 | 5.989147902368927 | 2.218910221686054 | 2.3724845009079587 |
| 8 | 1.8256978910504813 | 1.6595687411045457 | 0.8771929824561416 | 1.4082657744070513 |Cell death rate (%)
*
*
*
SC
KD
0
SC
KD
100
[μM]
Hep3B Normoxia
SC
KD
0
SC
KD
100
PA
HepG2 Normoxia
